# Dibromido(2,3-di-2-pyridyl­pyrazine-κ^2^ *N* ^2^,*N* ^3^)palladium(II)

**DOI:** 10.1107/S1600536811050525

**Published:** 2011-11-30

**Authors:** Kwang Ha

**Affiliations:** a School of Applied Chemical Engineering, The Research Institute of Catalysis, Chonnam National University, Gwangju 500-757, Republic of Korea

## Abstract

The Pd^II^ ion in the title complex, [PdBr_2_(C_14_H_10_N_4_)], is four-coordinated in a slightly distorted square-planar environment by the two pyridine N atoms of the chelating 2,3-di-2-pyridyl­pyrazine (dpp) ligand and two bromide anions. The pyridine rings are considerably inclined to the least-squares plane of the PdBr_2_N_2_ unit [maximum deviation = 0.080 (2) Å], making dihedral angles of 64.9 (1) and 66.4 (1)°. The pyrazine ring is perpendicular to the unit plane, with a dihedral angle of 89.0 (1)°. In the crystal, the complex mol­ecules are stacked in columns along the *a* axis and connected by C—H⋯Br hydrogen bonds, forming a helical chain along the *b* axis.

## Related literature

For related structures of [Pd*X*_2_(dpp)] (*X* = Cl, I), see: Ha (2011*a* ▶,*b* ▶). For related Pt, Pd and Mn complexes, see: Granifo *et al.* (2000 ▶); Armentano *et al.* (2003 ▶); Delir Kheirollahi Nezhad *et al.* (2008 ▶); Cai *et al.* (2009 ▶).
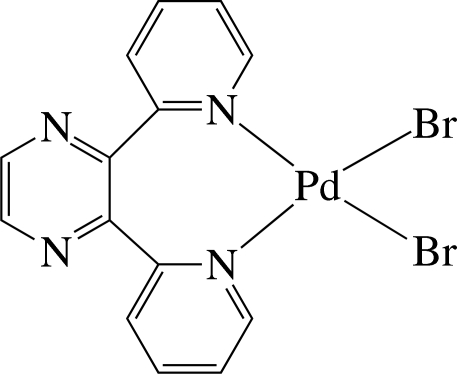


## Experimental

### 

#### Crystal data

[PdBr_2_(C_14_H_10_N_4_)]
*M* *_r_* = 500.48
Monoclinic, 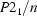

*a* = 8.515 (2) Å
*b* = 15.408 (4) Å
*c* = 11.941 (3) Å
β = 101.129 (5)°
*V* = 1537.3 (7) Å^3^
*Z* = 4
Mo *K*α radiation
μ = 6.40 mm^−1^
*T* = 200 K
0.26 × 0.11 × 0.10 mm

#### Data collection

Bruker SMART 1000 CCD diffractometer
Absorption correction: multi-scan (*SADABS*; Bruker, 2000 ▶) *T*_min_ = 0.689, *T*_max_ = 1.000
9366 measured reflections
2998 independent reflections
2384 reflections with *I* > 2σ(*I*)
*R*_int_ = 0.046

#### Refinement

*R*[*F* ^2^ > 2σ(*F* ^2^)] = 0.034
*wR*(*F* ^2^) = 0.086
*S* = 1.03
2998 reflections
190 parameters
H-atom parameters constrained
Δρ_max_ = 0.84 e Å^−3^
Δρ_min_ = −0.56 e Å^−3^

### 

Data collection: *SMART* (Bruker, 2000 ▶); cell refinement: *SAINT* (Bruker, 2000 ▶); data reduction: *SAINT*; program(s) used to solve structure: *SHELXS97* (Sheldrick, 2008 ▶); program(s) used to refine structure: *SHELXL97* (Sheldrick, 2008 ▶); molecular graphics: *ORTEP-3* (Farrugia, 1997 ▶) and *PLATON* (Spek, 2009 ▶); software used to prepare material for publication: *SHELXL97*.

## Supplementary Material

Crystal structure: contains datablock(s) global, I. DOI: 10.1107/S1600536811050525/zj2038sup1.cif

Structure factors: contains datablock(s) I. DOI: 10.1107/S1600536811050525/zj2038Isup2.hkl

Additional supplementary materials:  crystallographic information; 3D view; checkCIF report

## Figures and Tables

**Table:** 

Pd1—N3	2.029 (4)
Pd1—N4	2.031 (4)
Pd1—Br1	2.4183 (8)
Pd1—Br2	2.4288 (8)

**Table:** 

N3—Pd1—N4	87.44 (14)
Br1—Pd1—Br2	92.99 (3)

**Table 2:** Hydrogen-bond geometry (Å, °)

*D*—H⋯*A*	*D*—H	H⋯*A*	*D*⋯*A*	*D*—H⋯*A*
C11—H11⋯Br1^i^	0.95	2.88	3.670 (5)	141

Symmetry code: (i) 
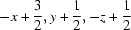
.

**Fig. 1.**
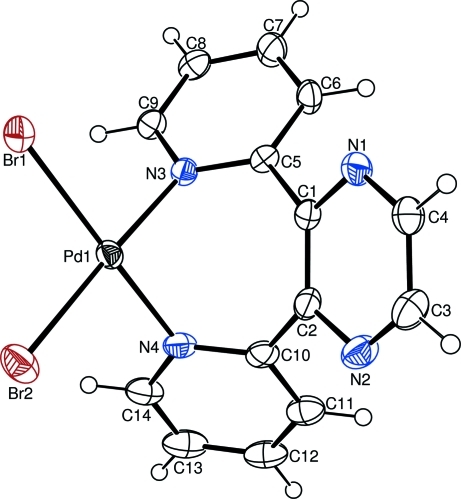
The structure of the title complex, with displacement ellipsoids drawn at the 40% probability level; H atoms are shown as small circles of arbitrary radius.

**Fig. 2.**
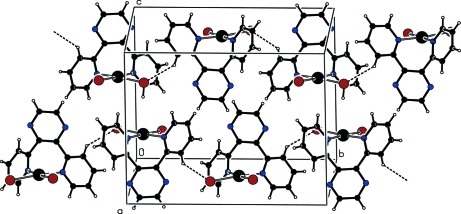
View of the hydrogen-bond interactions of the title complex. Hydrogen-bonds are drawn with dashed lines.

## supplementary crystallographic information

### Comment

Polypyridyl ligands have received considerable attention in coordination chemistry owing to the diverse coordination modes of the ligands (Granifo *et al.*, 2000; Armentano *et al.*, 2003; Delir Kheirollahi Nezhad *et al.*, 2008; Cai *et al.*, 2009).

The title complex, [PdBr_2_(dpp)] (dpp = 2,3-di-2-pyridylpyrazine, C_14_H_10_N_4_), is isomorphous with the previously reported analogous complexes [Pd*X*_2_(dpp)] (*X* = Cl, I) (Ha, 2011*a*,*b*). The Pd^II^ ion is four-coordinated in a slightly distorted square-planar environment by the two pyridine N atoms of the chelating dpp ligand and two bromide anions (Fig. 1). The coordination mode of the dpp ligand is similar to that found in the mononuclear Pt(II) and Pd(II) complexes [PtCl_2_(dpq)] (dpq = 2,3-di-2-pyridylquinoxaline) (Granifo *et al.*, 2000) and [MCl_2_(dcdpp)] (*M* = Pt, Pd; dcdpp = 2,3-dicyano-5,6-di-2-pyridylpyrazine) (Cai *et al.*, 2009).

The contributions to the distortion of square are the N3—Pd1—N4 chelate angle of 87.44 (14)° and Br—Br repelling, and therefore the *trans* axes are slightly bent [<Br1—Pd1—N4 = 173.69 (10)° and <Br2—Pd1—N3 = 177.29 (10)°]. The Pd—N and Pd—Br bond lengths are nearly equivalent, respectively (Table 1). In the crystal, the two pyridine rings are considerably inclined to the least-squares plane of the PdBr_2_N_2_ unit [maximum deviation = 0.080 (2) Å], making dihedral angles of 64.9 (1)° and 66.4 (1)°. The nearly planar pyrazine ring [maximum deviation = 0.022 (3) Å] is perpendicular to the unit plane with a dihedral angle of 89.0 (1)°. The dihedral angle between the two pyridine rings is 78.84 (1)°. The complex molecules are stacked in columns along the *a* axis and connected by C—H···Br hydrogen bonds, forming a helical chain along the *b* axis (Fig. 2 and Table 2). The hydrogen bonding mode is similar to that observed in the isotypic complex [PdI_2_(dpp)] (Ha, 2011*b*). By contrast, in the chloro analog [PdCl_2_(dpp)], two independent intermolecular C—H···Cl hydrogen bonds generate a layer structure extending parallel to the *ab* plane (Ha, 2011*a*). Along the *b* axis, successive molecules stack in the opposite directions. In the columns, numerous inter- and intramolecular π-π interactions between the six-membered rings are present, the shortest ring centroid-centroid distance being 3.849 (3) Å.

### Experimental

To a solution of K_2_PdBr_4_ (0.2513 g, 0.498 mmol) in MeOH (30 ml) was added 2,3-di-2-pyridylpyrazine (0.1188 g, 0.506 mmol) and refluxed for 3 h. The formed precipitate was separated by filtration, washed with MeOH, and dried at 50 °C, to give a yellow powder (0.2150 g). Crystals suitable for X-ray analysis were obtained by slow evaporation from a CH_3_NO_2_/acetone solution.

### Refinement

H atoms were positioned geometrically and allowed to ride on their respective parent atoms [C—H = 0.95 Å and *U*_iso_(H) = 1.2*U*_eq_(C)]. The highest peak (0.84 e Å^-3^) and the deepest hole (-0.56 e Å^-3^) in the difference Fourier map are located 0.88 Å and 0.69 Å from the atom Br1, respectively.

### Crystal data

**Table:**

[PdBr_2_(C_14_H_10_N_4_)]	*F*(000) = 952
*M**_r_* = 500.48	*D*_x_ = 2.162 Mg m^−^^3^
Monoclinic, *P*2_1_/*n*	Mo *K*α radiation, λ = 0.71073 Å
Hall symbol: -P 2yn	Cell parameters from 4302 reflections
*a* = 8.515 (2) Å	θ = 2.6–26.0°
*b* = 15.408 (4) Å	µ = 6.40 mm^−^^1^
*c* = 11.941 (3) Å	*T* = 200 K
β = 101.129 (5)°	Block, yellow
*V* = 1537.3 (7) Å^3^	0.26 × 0.11 × 0.10 mm
*Z* = 4	

### Data collection

**Table:**

Bruker SMART 1000 CCD diffractometer	2998 independent reflections
Radiation source: fine-focus sealed tube	2384 reflections with *I* > 2σ(*I*)
graphite	*R*_int_ = 0.046
φ and ω scans	θ_max_ = 26.0°, θ_min_ = 2.2°
Absorption correction: multi-scan (*SADABS*; Bruker, 2000)	*h* = −10→10
*T*_min_ = 0.689, *T*_max_ = 1.000	*k* = −18→15
9366 measured reflections	*l* = −14→14

### Refinement

**Table:**

Refinement on *F*^2^	Primary atom site location: structure-invariant direct methods
Least-squares matrix: full	Secondary atom site location: difference Fourier map
*R*[*F*^2^ > 2σ(*F*^2^)] = 0.034	Hydrogen site location: inferred from neighbouring sites
*wR*(*F*^2^) = 0.086	H-atom parameters constrained
*S* = 1.03	*w* = 1/[σ^2^(*F*_o_^2^) + (0.036*P*)^2^ + 0.1829*P*] where *P* = (*F*_o_^2^ + 2*F*_c_^2^)/3
2998 reflections	(Δ/σ)_max_ < 0.001
190 parameters	Δρ_max_ = 0.84 e Å^−^^3^
0 restraints	Δρ_min_ = −0.56 e Å^−^^3^

### Special details

**Table:**

Geometry. All e.s.d.'s (except the e.s.d. in the dihedral angle between two l.s. planes) are estimated using the full covariance matrix. The cell e.s.d.'s are taken into account individually in the estimation of e.s.d.'s in distances, angles and torsion angles; correlations between e.s.d.'s in cell parameters are only used when they are defined by crystal symmetry. An approximate (isotropic) treatment of cell e.s.d.'s is used for estimating e.s.d.'s involving l.s. planes.
Refinement. Refinement of *F*^2^ against ALL reflections. The weighted *R*-factor *wR* and goodness of fit *S* are based on *F*^2^, conventional *R*-factors *R* are based on *F*, with *F* set to zero for negative *F*^2^. The threshold expression of *F*^2^ > σ(*F*^2^) is used only for calculating *R*-factors(gt) *etc*. and is not relevant to the choice of reflections for refinement. *R*-factors based on *F*^2^ are statistically about twice as large as those based on *F*, and *R*- factors based on ALL data will be even larger.

### Fractional atomic coordinates and isotropic or equivalent isotropic displacement parameters (Å^2^)

**Table:**

	*x*	*y*	*z*	*U*_iso_*/*U*_eq_	
Pd1	0.52714 (4)	0.06214 (2)	0.31928 (3)	0.02593 (12)	
Br1	0.38710 (6)	−0.07475 (3)	0.31457 (4)	0.03824 (16)	
Br2	0.28059 (6)	0.14467 (4)	0.26892 (5)	0.04539 (18)	
N1	0.8184 (5)	0.0074 (3)	0.0802 (3)	0.0343 (9)	
N2	0.7670 (5)	0.1852 (3)	0.0770 (3)	0.0398 (10)	
N3	0.7370 (4)	−0.0038 (2)	0.3554 (3)	0.0252 (8)	
N4	0.6582 (5)	0.1732 (2)	0.3405 (3)	0.0307 (9)	
C1	0.8039 (5)	0.0504 (3)	0.1757 (4)	0.0259 (10)	
C2	0.7740 (5)	0.1390 (3)	0.1735 (4)	0.0301 (11)	
C3	0.7795 (6)	0.1421 (4)	−0.0176 (4)	0.0420 (13)	
H3	0.7716	0.1729	−0.0873	0.050*	
C4	0.8037 (6)	0.0538 (3)	−0.0161 (4)	0.0383 (12)	
H4	0.8101	0.0250	−0.0853	0.046*	
C5	0.8369 (5)	−0.0063 (3)	0.2804 (4)	0.0257 (10)	
C6	0.9677 (6)	−0.0606 (3)	0.2958 (4)	0.0348 (12)	
H6	1.0364	−0.0613	0.2419	0.042*	
C7	0.9980 (6)	−0.1141 (3)	0.3906 (4)	0.0403 (13)	
H7	1.0860	−0.1531	0.4017	0.048*	
C8	0.8991 (6)	−0.1098 (3)	0.4681 (4)	0.0354 (12)	
H8	0.9202	−0.1447	0.5349	0.042*	
C9	0.7696 (6)	−0.0552 (3)	0.4493 (4)	0.0295 (11)	
H9	0.7011	−0.0532	0.5033	0.035*	
C10	0.7647 (5)	0.1935 (3)	0.2749 (4)	0.0315 (11)	
C11	0.8591 (6)	0.2670 (3)	0.2963 (4)	0.0398 (13)	
H11	0.9325	0.2813	0.2485	0.048*	
C12	0.8459 (7)	0.3190 (3)	0.3869 (5)	0.0455 (14)	
H12	0.9106	0.3694	0.4031	0.055*	
C13	0.7370 (7)	0.2973 (4)	0.4548 (4)	0.0467 (15)	
H13	0.7255	0.3326	0.5179	0.056*	
C14	0.6474 (6)	0.2249 (3)	0.4294 (4)	0.0390 (13)	
H14	0.5735	0.2099	0.4765	0.047*	

### Atomic displacement parameters (Å^2^)

**Table:**

	*U*^11^	*U*^22^	*U*^33^	*U*^12^	*U*^13^	*U*^23^
Pd1	0.0282 (2)	0.0277 (2)	0.0233 (2)	0.00439 (15)	0.00855 (14)	0.00168 (14)
Br1	0.0316 (3)	0.0399 (3)	0.0447 (3)	−0.0018 (2)	0.0111 (2)	−0.0006 (2)
Br2	0.0450 (3)	0.0547 (4)	0.0378 (3)	0.0204 (3)	0.0114 (2)	0.0097 (2)
N1	0.044 (2)	0.032 (2)	0.030 (2)	0.0060 (19)	0.0149 (18)	0.0043 (18)
N2	0.050 (3)	0.034 (3)	0.036 (2)	0.000 (2)	0.009 (2)	0.010 (2)
N3	0.0271 (19)	0.024 (2)	0.026 (2)	−0.0015 (17)	0.0076 (16)	−0.0005 (16)
N4	0.044 (2)	0.022 (2)	0.026 (2)	0.0063 (18)	0.0077 (18)	0.0030 (16)
C1	0.022 (2)	0.030 (3)	0.027 (2)	−0.0020 (19)	0.0067 (18)	0.003 (2)
C2	0.029 (2)	0.033 (3)	0.030 (3)	−0.005 (2)	0.009 (2)	0.004 (2)
C3	0.053 (3)	0.043 (4)	0.030 (3)	−0.008 (3)	0.010 (2)	0.006 (2)
C4	0.046 (3)	0.042 (3)	0.031 (3)	0.003 (2)	0.017 (2)	0.000 (2)
C5	0.025 (2)	0.026 (3)	0.025 (2)	−0.003 (2)	0.0019 (18)	0.0001 (19)
C6	0.026 (2)	0.042 (3)	0.038 (3)	0.002 (2)	0.010 (2)	0.006 (2)
C7	0.029 (3)	0.042 (3)	0.049 (3)	0.004 (2)	0.006 (2)	0.011 (3)
C8	0.036 (3)	0.029 (3)	0.038 (3)	0.001 (2)	0.002 (2)	0.012 (2)
C9	0.034 (3)	0.031 (3)	0.023 (2)	−0.001 (2)	0.0033 (19)	0.001 (2)
C10	0.035 (3)	0.026 (3)	0.033 (3)	0.006 (2)	0.002 (2)	0.007 (2)
C11	0.040 (3)	0.031 (3)	0.045 (3)	−0.002 (2)	0.000 (2)	0.002 (2)
C12	0.053 (4)	0.024 (3)	0.052 (3)	−0.002 (2)	−0.010 (3)	−0.005 (3)
C13	0.071 (4)	0.034 (3)	0.030 (3)	0.010 (3)	−0.003 (3)	−0.002 (2)
C14	0.055 (3)	0.033 (3)	0.029 (3)	0.012 (3)	0.009 (2)	0.003 (2)

### Geometric parameters (Å, °)

**Table:**

Pd1—N3	2.029 (4)	C4—H4	0.9500
Pd1—N4	2.031 (4)	C5—C6	1.376 (6)
Pd1—Br1	2.4183 (8)	C6—C7	1.384 (7)
Pd1—Br2	2.4288 (8)	C6—H6	0.9500
N1—C4	1.339 (6)	C7—C8	1.368 (7)
N1—C1	1.345 (6)	C7—H7	0.9500
N2—C3	1.333 (6)	C8—C9	1.371 (7)
N2—C2	1.346 (6)	C8—H8	0.9500
N3—C5	1.349 (5)	C9—H9	0.9500
N3—C9	1.357 (6)	C10—C11	1.385 (7)
N4—C10	1.344 (6)	C11—C12	1.368 (7)
N4—C14	1.345 (6)	C11—H11	0.9500
C1—C2	1.388 (6)	C12—C13	1.385 (8)
C1—C5	1.506 (6)	C12—H12	0.9500
C2—C10	1.488 (6)	C13—C14	1.353 (7)
C3—C4	1.374 (7)	C13—H13	0.9500
C3—H3	0.9500	C14—H14	0.9500
			
N3—Pd1—N4	87.44 (14)	C6—C5—C1	118.7 (4)
N3—Pd1—Br1	88.74 (10)	C5—C6—C7	119.3 (5)
N4—Pd1—Br1	173.69 (10)	C5—C6—H6	120.3
N3—Pd1—Br2	177.29 (10)	C7—C6—H6	120.3
N4—Pd1—Br2	91.01 (11)	C8—C7—C6	118.9 (5)
Br1—Pd1—Br2	92.99 (3)	C8—C7—H7	120.6
C4—N1—C1	117.1 (4)	C6—C7—H7	120.6
C3—N2—C2	117.7 (4)	C7—C8—C9	120.0 (4)
C5—N3—C9	118.6 (4)	C7—C8—H8	120.0
C5—N3—Pd1	121.0 (3)	C9—C8—H8	120.0
C9—N3—Pd1	119.9 (3)	N3—C9—C8	121.5 (5)
C10—N4—C14	118.5 (4)	N3—C9—H9	119.2
C10—N4—Pd1	122.7 (3)	C8—C9—H9	119.2
C14—N4—Pd1	118.6 (4)	N4—C10—C11	121.1 (5)
N1—C1—C2	121.0 (4)	N4—C10—C2	119.4 (4)
N1—C1—C5	112.6 (4)	C11—C10—C2	119.4 (4)
C2—C1—C5	126.1 (4)	C12—C11—C10	119.4 (5)
N2—C2—C1	120.9 (4)	C12—C11—H11	120.3
N2—C2—C10	113.4 (4)	C10—C11—H11	120.3
C1—C2—C10	125.3 (4)	C11—C12—C13	119.2 (5)
N2—C3—C4	121.2 (5)	C11—C12—H12	120.4
N2—C3—H3	119.4	C13—C12—H12	120.4
C4—C3—H3	119.4	C14—C13—C12	118.7 (5)
N1—C4—C3	121.9 (5)	C14—C13—H13	120.6
N1—C4—H4	119.0	C12—C13—H13	120.6
C3—C4—H4	119.0	N4—C14—C13	123.0 (5)
N3—C5—C6	121.7 (4)	N4—C14—H14	118.5
N3—C5—C1	119.6 (4)	C13—C14—H14	118.5
			
N4—Pd1—N3—C5	−71.4 (3)	N1—C1—C5—C6	44.7 (6)
Br1—Pd1—N3—C5	113.6 (3)	C2—C1—C5—C6	−130.3 (5)
N4—Pd1—N3—C9	116.8 (3)	N3—C5—C6—C7	−0.1 (7)
Br1—Pd1—N3—C9	−58.2 (3)	C1—C5—C6—C7	−178.9 (4)
N3—Pd1—N4—C10	61.5 (4)	C5—C6—C7—C8	−1.7 (8)
Br2—Pd1—N4—C10	−116.3 (3)	C6—C7—C8—C9	2.1 (8)
N3—Pd1—N4—C14	−113.0 (3)	C5—N3—C9—C8	−1.1 (6)
Br2—Pd1—N4—C14	69.3 (3)	Pd1—N3—C9—C8	170.9 (3)
C4—N1—C1—C2	0.4 (6)	C7—C8—C9—N3	−0.7 (7)
C4—N1—C1—C5	−174.9 (4)	C14—N4—C10—C11	−1.5 (7)
C3—N2—C2—C1	4.2 (7)	Pd1—N4—C10—C11	−175.9 (3)
C3—N2—C2—C10	178.0 (4)	C14—N4—C10—C2	−177.6 (4)
N1—C1—C2—N2	−3.5 (7)	Pd1—N4—C10—C2	7.9 (6)
C5—C1—C2—N2	171.1 (4)	N2—C2—C10—N4	129.0 (4)
N1—C1—C2—C10	−176.5 (4)	C1—C2—C10—N4	−57.6 (6)
C5—C1—C2—C10	−1.9 (7)	N2—C2—C10—C11	−47.3 (6)
C2—N2—C3—C4	−1.9 (8)	C1—C2—C10—C11	126.2 (5)
C1—N1—C4—C3	1.9 (7)	N4—C10—C11—C12	1.2 (7)
N2—C3—C4—N1	−1.1 (8)	C2—C10—C11—C12	177.4 (4)
C9—N3—C5—C6	1.5 (6)	C10—C11—C12—C13	−0.6 (8)
Pd1—N3—C5—C6	−170.4 (3)	C11—C12—C13—C14	0.3 (8)
C9—N3—C5—C1	−179.7 (4)	C10—N4—C14—C13	1.1 (7)
Pd1—N3—C5—C1	8.4 (5)	Pd1—N4—C14—C13	175.8 (4)
N1—C1—C5—N3	−134.1 (4)	C12—C13—C14—N4	−0.5 (8)
C2—C1—C5—N3	50.8 (6)		

### Hydrogen-bond geometry (Å, °)

**Table:**

*D*—H···*A*	*D*—H	H···*A*	*D*···*A*	*D*—H···*A*
C11—H11···Br1^i^	0.95	2.88	3.670 (5)	141.

Symmetry codes: (i) −*x*+3/2, *y*+1/2, −*z*+1/2.
